# Based on a Decision Tree Model for Exploring the Risk Factors of Smartphone Addiction Among Children and Adolescents in China During the COVID-19 Pandemic

**DOI:** 10.3389/fpsyt.2021.652356

**Published:** 2021-06-08

**Authors:** Li Duan, Juan He, Min Li, Jiali Dai, Yurong Zhou, Feiya Lai, Gang Zhu

**Affiliations:** ^1^Department of Psychiatry, The First Affiliated Hospital of China Medical University, Shenyang, China; ^2^Central Laboratory, The First Affiliated Hospital of China Medical University, Shenyang, China

**Keywords:** decision tree model, smartphone addiction, COVD-19, children, adolescents

## Abstract

**Background:** Smartphone addiction has emerged as a major concern among children and adolescents over the past few decades and may be heightened by the outbreak of COVID-19, posing a threat to their physical and mental health. Then we aimed to develop a decision tree model as a screening tool for unrecognized smartphone addiction by conducting large sample investigation in mainland China.

**Methods:** The data from cross-sectional investigation of smartphone addiction among children and adolescents in mainland China (*n* = 3,615) was used to build models of smartphone addiction by employing logistic regression, visualized nomogram, and decision tree analysis.

**Results:** Smartphone addiction was found in 849 (23.5%) of the 3,615 respondents. According to the results of logistic regression, nomogram, and decision tree analyses, Internet addiction, hours spend on smartphone during the epidemic, levels of clinical anxiety symptoms, fear of physical injury, and sex were used in predictive model of smartphone addiction among children and adolescents. The C-index of the final adjusted model of logistic regression was 0.804. The classification accuracy, sensitivity, specificity, positive predictive value, negative predictive value, and AUC area of decision tree for detecting smartphone addiction were 87.3, 71.4, 92.1, 73.5, 91.4, and 0.884, respectively.

**Conclusions:** It was found that the incidence of smartphone addiction among children and adolescents is significant during the epidemic. The decision tree model can be used to screen smartphone addiction among them. Findings of the five risk factors will help researchers and parents assess the risk of smartphone addiction quickly and easily.

## Introduction

The use of personal mobile devices connected to the Internet has become commonplace in the contemporary society. With the advent of the fifth-generation (5G) mobile networks and the continuous innovation of scientific technology, smartphones with many functions have become an integral part of our daily lives. The 44th Statistical Report on China's Internet Development, released by the China Internet Network Information Center (1) has stated that the number of Chinese netizens (people involved in online communities and users of Internet) under 18 years old hit 175 million as of June 2019, with an Internet penetration rate of 93.1%. Nearly 99.1% of them connect to the Internet via smartphones, indicating that smartphones have overtaken computers as the most commonly used devices to access the Internet (2). With the increasing pervasiveness of the smartphone and Internet in the adolescent population, the age of minors who were exposed to the Internet is becoming increasingly younger. Among them, the proportion of primary-, middle-, and high-school students using smartphones for the first time account for 23.8, 38.1, and 18.0%, respectively (1). Moreover, sensation/novelty seeking (3) and rapid psychological and intellectual maturation (4) make this age group particularly susceptible to smartphone attraction.

With the development and improvement in the Diagnostic and Statistical Manual of Mental Disorders (DSM-5) and the International Classification of Disease (11 revision; ICD-11), researchers have introduced non-substance addiction as a psychiatric diagnosis, and have become increasingly concerned about gaming disorders due to their addictive behaviors (5, 6). Smartphone/Internet addiction, recognized as a non-substance addiction, has attracted increasing attention from educators, health personnel, and the popular media (7–9) due to its undesired and disadvantageous consequences for users. Such consequences include academic failures, physical and mental health problems, and sleep disturbances caused by excessive, uncontrolled, or inappropriate use of the smartphone (2, 10, 11). In the past, studies on smartphone addiction were mostly based on Internet addiction (12, 13). Multi-dimensional factors, such as poor peer relationships, low self-esteem, personality traits, and mental health status, can have an impact on the behavioral disorders of individuals with smartphone/Internet addiction (14–17). Moreover, psychological interventions, such as cognitive behavioral therapy (CBT), sandplay therapy and educational training programming, were recognized as the main effective approaches to reduce the addiction severity (18). However, different studies on the effectiveness of CBT are controversial, and further clinical validation researches are needed (19).

In December 2019, the novel coronavirus disease (COVID-19) was first reported in Wuhan, Hubei Province, China, spreading readily to other regions in mainland China and subsequently to more than 210 countries globally. According to data released by the United Nations Educational Scientific and Cultural Organization (UNESCO), the pandemic is impacting close to 363 million students worldwide from the pre-primary to tertiary level (20). Among them, one in five students is being kept out of school due, and an additional one in four is being kept out of higher education establishments (20). In China, more than 220 million children and adolescents began online study courses at home instead of conventional teaching models at school due to the impact of the coronavirus (21). However, given unstable network signals, the imperfect management of online teaching and examination, and higher risk of visual loss caused by cumulative effect of blue light emitted from various electronic devices (22, 23), online teaching is not only difficult to meet the learning needs of students, but it also may further aggravate mental distress. Additionally, prolonged quarantined at home and a heightened risk of witnessing or suffering violence and abuse can lead them to difficulties in concentration, as well as irritability, restlessness, and nervousness (24), and mental health status has been identified as a prominent factor for smartphone addiction (16).

At present, the epidemic is still spreading in some countries, and it will take a long time to completely eliminate the coronavirus in the future. Traditional face-to-face teaching is still at great risks, and the combination way of online and offline has become the main method adopted by educational institutions. Thus, we investigated on smartphone addiction among children and adolescents, analyzed its prevalence and developed a decision tree model as a screening tool for unrecognized smartphone addiction during the specific COVID-19 pandemic period.

It aimed to provide scientific fundaments for other countries to screen and cope with smartphone addiction among children and adolescents during the tough period of global pandemic.

## Methods

### Study Population

A large sample questionnaire survey was administered between February 25 to April 25, 2020. Considering the difficulties caused by the epidemic to field investigation, we used the program “Questionnaire Star (https://www.wjx.cn/)” to distribute and retrieve questionnaires online. The program is well-recognized as a professional online survey tool, and allows researchers to create, distribute, and analyze online surveys easily and efficiently. By employing a convenience cluster sampling method, children and adolescents ranging from Grade 1 in primary school to Grade 3 in high school (aged 7–18 years) were recruited across mainland China.

Initially, respondents of 3,706 children aged from 7 to 12 years and adolescents aged from 13 to 18 years were recruited using a convenience sample approach with the help of directors in the Education Bureau and schools. They assigned tasks to teachers, who were responsible for distributing e-questionnaires and a manual of procedures to a WeChat group that included teachers, students, and parents. For junior students from primary school, they needed to understand contents of items with the help of their guardians, and to fill out questionnaires according to their own judgement, while older adolescents could complete them independently. Additionally, the guardians who interpreted the contents of the items should meet the criteria: living together with the subjects (convenient to observe whether they understand the content of the item through their expression); have enough time to communicate with the subjects; without any communication barriers; responsible for taking care of their daily life and tutoring their study tasks. All the respondents were able to submit and repost the questionnaires directly after filling it out online.

After eliminating 91 respondents for incomplete, invalid, or missing data, the final sample consisted of 3,615 respondents from 18 provinces/municipalities in mainland China, resulting in a response rate of 97.54%.

### Measurements

- **Personal information form**. The authors designed this part of the questionnaire, which consisted of questions regarding social-demographic characteristics (e.g., sex, age, and place of residence), as well as Internet usage habits, goals, and other COVID-related information (e.g., family members involved in anti-epidemic work, hours spend on smartphone per day before/during the epidemic, and purpose of smartphone usage before/during the epidemic).
- **Short Version of Smartphone Addiction Scale (SAS-SV)** is a 10-item self-administered scale of SV-SAS which was developed to assess a high-risk group of adolescents with smartphone excessive use or addiction using a 6-point Likert scale from 1 (not at all) to 6 (very much) (25, 26). The internal consistency of the test was verified with a Cronbach's α of 0.911 in adolescents in Korea (25), 0.79 in adolescents and young adults in Italy (27) and 0.86 in adolescents in China (15). For this study, after conducting investigation on school students using SV-SAS, the Cronbach's α value was 0.916, which further demonstrated that this measurement had a good reliability. In references to the previous studies, the cut-off value of this scale was defined by sex, specifically 31 for female and 33 for male, respectively (25, 28).
- **The Internet Addiction Scale (IAS)** was designed for screening Internet addicts (≥70), possible Internet addicts (40–69), and non-addict (≤30) referring to the diagnostic criteria (DSM-IV-TR) of pathological gaming and the degree of preoccupation and compulsives to go online (29). In this study, the threshold value of Internet addiction was set as the scores of 70 or higher by employing the sum of all the 20 items with a 5-point Likert scale range from 1 (never) to 5 (always).
- **Spence Child Anxiety Scale** (SCAS) was first designed by Spence in 1997 (30), and which was then further refined by Zhao and Wang et al. to construct a self-rating scale for children and adolescents (31). This scale comprises six subscales (including separation anxiety, physical injury fear, social phobia, panic disorder, obsessive disorder, generalized anxiety) with a total of 44 items that evaluate anxiety symptoms among children and adolescents, with a 4-point Likert scale ranging from 0 (never) to 3 (always). Scores of the total scale each subscale are calculated by totaling the responses.
- **Children's Depression Inventory** (CDI) was initially developed and further modified by Kovacs et al. (32, 33), and measures depression symptoms among children and adolescents aged 7–17 years. Prior work has demonstrated that the CDI has satisfactory reliability and validity in the Chinese population (34), and can be divided into three types of depressive symptom-screening groups: clinical depressive symptom (≥19), subclinical depression (12–18), and normal (≤12) (35).
- **Coping Style Scale (CSS)** was developed by Chen et al. in 2000 based on the theory of social interaction and self-regulation with a sample of Chinese middle-school students (36, 37). It has 36 items in a Likert-style scale, ranging from 1 (never) to 4 (often), and asks respondents to rate their competence in coping with stress from either a problem-focused perspective (including solving problems, seeking social support, and positive rationalizations) or emotion-focused perspective (including endurance, avoidance, expressing emotions, and fantasy/denial). Higher scores indicate better ability to cope with stress (36).

### Ethics Statement

This study involving participants of children and adolescents in mainland China were reviewed and approved the Ethics Committee of the First Affiliated Hospital of China Medical University (No. 2020-202-2). Prior to filling out the survey, all of the anonymous volunteer participants and their guardians were informed of the purpose and significance of the study in detail, and freely made the decision to participate in or not.

### Statistical Analysis

Statistical analyses were performed with SPSS (Version 18.0, SPSS Inc., Chicago) for Windows and the R version 3.6.3 (http://www.r-project.org). Frequencies (percentages) and means (standard deviations, SDs) were used to analyze personal information, levels of the respondents' depression, as well as continuous variables of anxiety symptoms and scores relating to problem/emotion-focused coping styles. Chi-square tests were conducted to investigate differences in the personal information, and we constructed the categorical variable of the presence of depression symptoms to compare between the smartphone addiction and non-addiction groups.

We then assessed the association between outcome variables (the reported level of smartphone addiction) and potential predictors (including demographics, COVID-related variables, as well as levels of anxiety, and coping style) employing bivariate logistic regression analyses, while adjusting for other identified explanatory variables with a *P-*value less than or equal to 0.05. Moreover, we further simplified the complex logistic regression model into a visualized nomogram, and measured its discrimination (the model's ability to distinguish among participants whether they developed smartphone addiction or not, as indicated by modification of Harrell's C-index to accommodate censoring) employing R and calibration (agreement between observed and predicted proportions of participants with smartphone addiction) employing calibration plots.

Afterwards, a decision tree, as a non-linear discrimination method, which was considered to be one of the most popular approaches for representing classifier in the field of statistics, machine learning, data mining, and medicine (38). It can be used to build models by splitting the sample into progressively smaller subgroups. Then, the specific procedure is iterative at each branch of the tree, and the independent variables which have the most significant relationship with the dependent/outcome variable are selected step by step by employing a specific criterion (39). That is, smartphone addiction among children and adolescents presents or absents was set as the target outcome variable in this study. Starting at the root of the decision tree, data were split into two groups that best separated the target classes, and repeated this procedure for each of the child-node until all variables were assigned to high or low risk group. The decision rules also provide specific information about risk factors on the basis of rule induction. In order to derive a reliable conclusion, 2,000 subjects were randomly selected from all participants and all smartphone addiction subjects were used to develop a decision tree model, and other remaining data were used to validate it internally. Then the classification accuracy, sensitivity, specificity, positive/negative predictive value and area under the curve (AUC) were calculated to further test the accuracy of this model.

## Results

We analyzed data from 3,615 participants (49.8% males and 50.2%females) with a mean age of 14.57 (SD = 2.1, range = 7–18 years). In terms of the residential areas, 0.6% (23/3615) of the participants were from Hubei Province, the hardest-hit city of the pandemic in China. As expected, 80% (2894/3615) of subjects reported smartphone possession. Further analysis demonstrated that 3.0% (109/3615) of them already owned smartphones by the age of six, while the penetration rates of smartphone for children and adolescents were 40.1% (1448/3615) and 18.3% (661/3615), respectively. During the outbreak, the number of respondents using smartphones for more than 5 h a day rose from 85 prior to the outbreak to 288 during the pandemic period. The main purpose of using smartphones in both periods was to study, but the proportion was much higher (78.5%) during the epidemic than before (57.0%). Compared with younger children (24.8%), older adolescents (23.3%) were more likely to become addicted to smartphones, and the overall prevalence rate of smartphone addiction among all of the respondents was 23.5% (813/3615). Other demographics and COVID-related characteristics of the samples are presented in Tables 1, 2.

**Table 1:** Frequencies and chi-square test of smartphone addiction and non-addicts on social-demographic characteristics (*N* = 3,615).

**Variables**	**Total *N* (%)**	**Smartphone addiction** ***N*** **(%)**		**X^**2**^**
		**Yes (*N =* 1,237)**	**No (*N* = 2,378)**	
**Sex**				
Male	1,799 (49.8)	366 (20.3)	1,433 (79.7)	19.659^**^
Female	1,816 (50.2)	483 (26.6)	1,333 (73.4)	
**Age (years)**				
7–12	351 (9.7)	87 (24.8)	264 (75.2)	0.366
13–18	3,264 (90.3)	762 (23.3)	2,502 (76.7)	
**Residential areas**				
Hubei Province	23 (0.6)	6 (5.4)	17 (73.9)	0.087
Others	3,592 (99.4)	843 (23.5)	2,749 (76.5%)	
**Region**				
Urban	1,781 (49.3)	428 (24.0)	1,353 (76.0)	1.799
Town	384 (10.6)	80 (20.8)	304 (79.2)	
Rural	1,450 (40.1)	341 (23.5)	1,109 (76.5)	
**Only child status**				
Yes	1,775 (49.1)	417 (23.5)	1,358 (76.5)	0.001
No	1,840 (50.9)	432 (23.5)	1,408 (76.6)	
**Family status**^**†**^				
Nuclear family	2,459 (68.0)	557 (22.7)	1,902 (77.3)	3.071
Extended family	887 (24.5)	225 (25.4)	662 (74.6)	
Single-parent family	73 (2.0)	19 (26.0)	54 (74.0)	
Etc. (e.g., step-family)	196 (5.4)	48 (24.5)	148 (75.5)	
**Education level**				
Primary school	211 (5.8)	55 (26.1)	156 (73.9)	1.006
Secondary school	2,041 (56.5)	471 (23.1)	1,570 (76.9)	
High school	1,363 (37.7)	323 (23.7)	1,040 (76.3)	
**Types of the school**^**‡**^				
Ordinary	1,634 (45.2)	350 (22.0)	1,284 (78.0)	7.081^**^
Key	1,981 (54.8)	499 (24.7)	1,482 (75.3)	
**Age at possessing smartphones**				
Yes, before 6 years old	109 (3.0)	94 (86.2)	15 (13.8)	14.884^*^
Yes, during 7–12 years old	1,448 (40.1)	1,112 (76.8)	336 (23.2)	
Yes, during 13–18 years old	1,397 (38.6)	1,135 (81.2)	262 (18.8)	
No^Δ^	661 (18.3)	503 (76.1)	158 (23.9)	
**Have electronic devices**				
Only have smartphone	1,725 (47.7)	1,372 (79.5)	353 (20.5)	44.830^**^
Have smartphone and other devices	1,169 (32.3)	967 (82.7)	202 (17.3)	
Have other devices without smartphone	296 (8.2)	203 (68.6)	93 (31.4)	
No^**Δ**^	425 (11.8)	302 (71.1)	123 (28.9)	

† *Nuclear family denotes living with parents, and extended family represents living with parents and grandparents*.

Δ *“No” represents that respondents do not possess smartphones or electronic devices independently or share them with other siblings. However, they still have the opportunity to access to the mobile network through smartphones of their caregivers or friends*.

‡ *Compared with other schools, key schools are ranked at the top of their regional rankings in terms of their comprehensive strength. Moreover, key schools often select the best students based on their entrance examination and interview scores, while ordinary schools take the rest as their main source of students*.

*SAS-SV, Short version of the Smartphone Addiction Scale*.

* *P < 0.05*;

** *P < 0.01*.

**Table 2:** The impact of reported COVID-19 related information and clinical depressive symptoms on smartphone addiction (*N* = 3,615).

**Variables**	**Total *N* (%)**	**Smartphone addiction** ***N*** **(%)**		**X^**2**^**
		**Yes (*N =* 1,237)**	**No (*N* = 2,378)**	
**Family member involved in anti-epidemic work**				
Yes	167 (4.6)	70 (41.9)	97 (58.1)	4.609^*^
No	3,448 (95.4)	1,167 (33.8)	2,281 (66.2)	
**Occupation of the father who involved in anti-epidemic work**				
Medical personal	20 (12.0)	7 (35.0)	13 (65.0)	0.749
Non-medical-staff	147 (88.0)	38 (25.9)	109 (74.1)	
**Occupation of the mother who involved in anti-epidemic work**				
Medical personal	31 (18.6)	10 (32.3)	21 (67.7)	0.546
Non-medical-staff	136 (81.4)	35 (25.7)	101 (74.3)	
**Family member or friend infected with coronavirus**				
Yes	16 (0.4)	4 (25.0)	12 (75.0)	0.063
No	3,599 (99.6)	845 (23.5)	2,754 (76.5)	
**Concern about the epidemic**				
Very concerned	2,109 (58.4)	480 (22.8)	1,629 (77.2)	0.619
Concerned	1,182 (32.7)	287 (24.3)	895 (75.7)	
Average	301 (8.3)	79 (26.2)	222 (73.8)	
Not concerned	15 (0.4)	4 (26.7)	11 (73.3)	
Very unconcerned	8 (0.2)	1 (12.5)	7 (87.5)	
**Implementation of the precaution and control measures**				
Strictly enforced	3,396 (94.9)	801 (23.6)	2,595 (76.4)	2.462
Sometimes	202 (5.6)	42 (20.8)	160 (79.2)	
Occasionally	13 (0.4)	5 (38.5)	8 (61.5)	
Never	4 (0.1)	1 (25.0)	3 (75.0)	
**Learning affected by the epidemic**				
Yes	1,978 (54.7)	443 (22.4)	1,535 (77.6)	2.883
No	1,637 (45.3)	406 (24.8)	1,231 (75.2)	
**Graduation affected by the epidemic**				
Yes	1,286 (35.1)	287 (22.3)	999 (77.7)	1.516
No	2,329 (64.4)	562 (24.1)	1,767 (75.9)	
**Hours spend on smartphone per day before the epidemic**				
≤ 1 h	1,258 (34.8)	296 (23.5)	962 (76.5)	0.026
1–3 h	1,413 (39.1)	333 (23.6)	1,080 (76.4)	
3–5 h	578 (16.0)	135 (23.4)	443 (76.6)	
≥5 h	366 (10.1)	85 (23.2)	281 (76.8)	
**Hours spend on smartphone per day during the epidemic**				
≤ 1 h	396 (11.0)	79 (19.9)	317 (80.1)	10.659^*^
1–3 h	1,117 (30.9)	264 (23.6)	853 (76.4)	
3–5 h	1,016 (28.1)	218 (21.5)	798 (78.5)	
≥5 h	1,086 (30.0)	288 (26.5)	798 (73.5)	
**Purpose of smartphone usages before the epidemic**				
Study	2,059 (57.0)	1,528 (74.2)	531 (25.8)	84.604^**^
Chatting	489 (13.5)	442 (90.4)	47 (9.6)	
Watching videos	264 (7.3)	229 (86.7)	35 (13.3)	
Surfing on Internet	467 (12.9)	361 (77.3)	106 (22.7)	
Play games online	254 (7.0)	219 (86.2)	35 (13.8)	
Etc.	82 (2.3)	65 (79.3)	17 (20.7)	
**Purpose of smartphone usages during the epidemic**				
Study	2,837 (78.5)	2,183 (76.9)	654 (23.1)	26.895^**^
Chatting	222 (6.1)	193 (86.9)	29 (13.1)	
Watching videos	144 (4.0)	124 (86.1)	20 (13.9)	
Surfing on the Internet	211 (5.8)	170 (80.6)	41 (19.4)	
Play games online	149 (4.1)	130 (87.2)	19 (12.8)	
Etc.	52 (1.4)	44 (84.6)	8 (15.4)	
**Willingness to engage in medical profession**				
Always	1,590 (44.0)	335 (21.1)	1,255 (78.9)	11.823^**^
A little uncertain after the epidemic	278 (7.7)	76 (27.3)	202 (72.7)	
Very willingly after the epidemic	678 (18.7)	159 (23.5)	519 (76.5)	
Never	1,069 (29.6)	281 (26.3)	788 (73.7)	
**Clinical depressive symptoms**				
Non-depressed	2,121 (58.7)	490 (23.1)	1,631 (76.9)	0.513
Subclinical depression	722 (20.0)	176 (24.4)	546 (75.6)	
Clinical depression	772 (21.3)	183 (23.7)	589 (76.3)	

* *P < 0.05*;

** *P < 0.01*.

We classified 849 subjects, including 366 males and 483 females, who scored 19 or above on the SAS-SV as smartphone addicts. According to chi-square analysis (Tables 1, 2), smartphone addictive respondents were more likely to be female (X^2^ = 19.659, *P* < 0.01), study in key schools (X^2^ = 7.081, *P* < 0.01), possess smartphones at an earlier age (X^2^ = 14.884, *P* < 0.01), have smartphones or other electronic devices (X^2^ = 44.830, *P* < 0.01), family members involved in pandemic work (X^2^ = 4.609, *P* < 0.05), spend more time per day on smartphone during the pandemic (X^2^ = 10.659, *P* < 0.05), the main purpose of smartphone usages before (X^2^ = 84.604, *P* < 0.01) and during (X^2^ = 26.895, *P* < 0.01) the epidemic, as well as willingness to engage in medical profession (X^2^ = 11.823, *P* < 0.01).

Table 3 shows the means and SDs, as well as the results of a *t-*test that examined the influence of continuous variables (e.g., coping styles, anxiety symptoms, and its six subscales) on smartphone addiction. The results revealed that the overall level of anxiety symptoms and its six subscales of all of the children and adolescents with smartphone addiction were significantly higher than those of non-addicts (*P* < 0.01). However, when examined as a function coping style, we found that respondents who were primarily emotion focused in their coping scores were significantly higher than those of non-addicts (*P* < 0.05), while those who adopted a more problem-focused coping style scored significantly lower than those of non-addicts (*P* < 0.01). That is, the ability of smartphone addicts to cope with stress was significantly lower than that of non-addicts.

**Table 3:** Means, standard deviations on anxiety and coping style for smartphone addiction and non-addiction.

**Variables**	**Total**	**Smartphone addiction**		***t***
		**Yes (*N* = 1,237)**	**No (*N* = 2,378)**	
**Anxiety symptoms**	28.76 ± 19.22	38.78 ± 21.08	25.68 ± 17.50	18.148^**^
Separation anxiety	4.25 ± 3.45	5.69 ± 3.74	3.80 ± 3.22	14.394^**^
Physical injury fear	4.15 ± 3.18	5.10 ± 3.23	3.86 ± 3.10	10.012^**^
Social phobia	6.11 ± 4.00	8.07 ± 4.33	5.51 ± 3.70	16.958^**^
Panic disorder	4.66 ± 4.93	6.92 ± 5.62	3.97 ± 4.47	15.752^**^
Obsessive disorder	4.45 ± 3.78	6.09 ± 4.20	3.94 ± 3.50	14.925^**^
Generalized anxiety	5.13 ± 3.60	6.91 ± 3.93	4.59 ± 3.30	17.100^**^
**Coping style**				
Problem-focused coping style	53.26 ± 11.61	51.74 ± 13.23	53.67 ± 11.08	−4.247^**^
Emotion-focused coping style	37.09 ± 9.80	37.99 ± 11.04	36.89 ± 9.40	2.890^*^

*SD, Standard Deviation*.

* *P < 0.05*;

** *P < 0.01*.

Logistic regression analysis identified four factors as being significantly associated with an increased level of smartphone addiction in children and adolescents: hours spend on smartphone per day during the epidemic (adjusted OR 1.544, 95%CI 1.402–1.703), total scores of SCAS (adjusted OR 1.206, 95%CI 1.152–1.264), and tendency to adopt an emotion-focused copying style (adjusted OR 1.059, 95%CI 1.047–1.071), and Internet addiction (adjusted OR 21.438, 95%CI 12.418–34.387). In contrast, other fours variables, including sex (adjusted OR 0.565, 95%CI 0.465–0.686), willingness to engage in medical profession (adjusted OR 0.928, 95%CI 0.863–0.997), fear of physical injury (adjusted OR 0.656, 95%CI 0.527–0.816), problem-focused coping style (adjusted OR 0.987, 95%CI 0.977–0.996) were found to be significantly associated with decreased levels of smartphone addiction.

**Figure 1:**
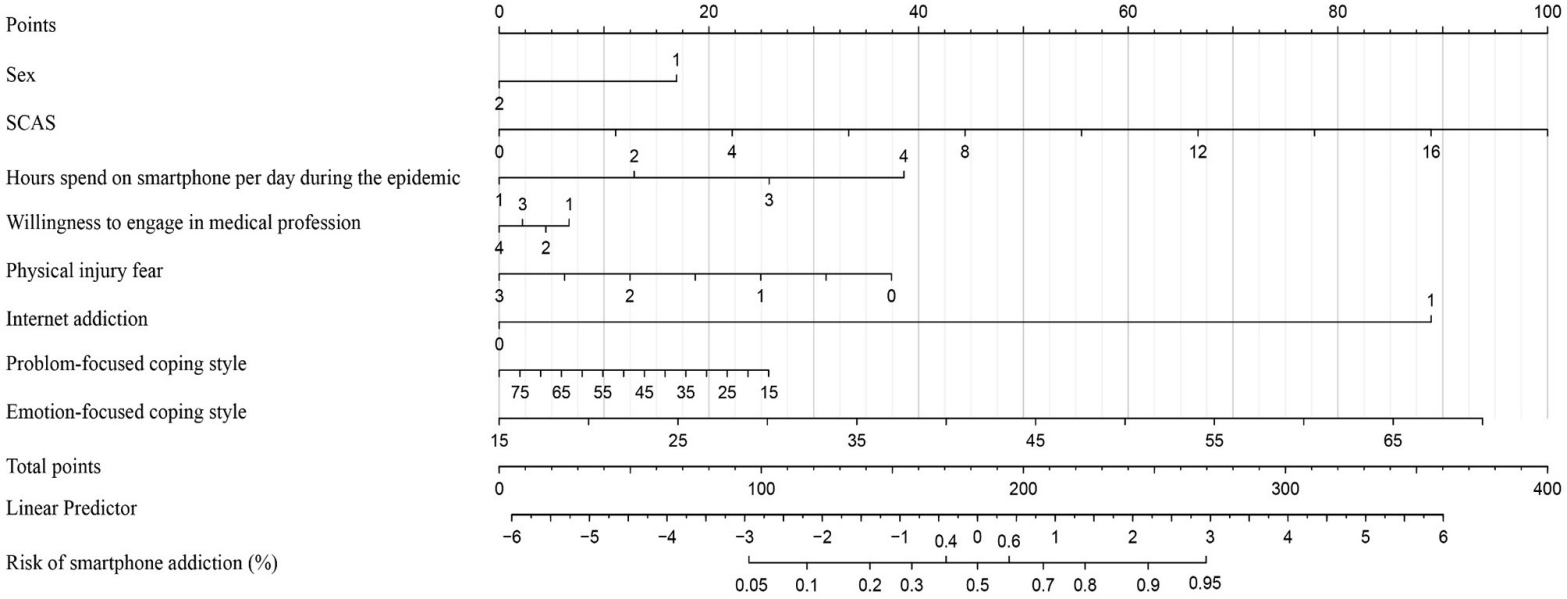
Nomogram for prediction the risk of smartphone addiction. Points for sex, levels of clinical anxiety symptoms (total scores of SCAS), hours spend on smartphone per day during epidemic, willingness to engage in medical profession, physical injury fear, levels of Internet addiction and emotion/problem-focused coping style can be obtained by calibrating with the point caliper, and then combined to obtain a total score that can be calibrated with the cumulative risk of smartphone addiction (%). The assignment values of each classified variable were: sex, 1 = female; 2 = male; hours spend on smartphone per day during the epidemic, 1(≤1 h)−4 (≥5 h); willingness to engage in medical profession, 1 = never, 2 = very willingly after the epidemic, 3 = a little uncertain after the epidemic, 4 = always; physical injury fear, 0 (never)−3 (always); Internet addiction, 1 = yes, 2 = no.

**Figure 2:**
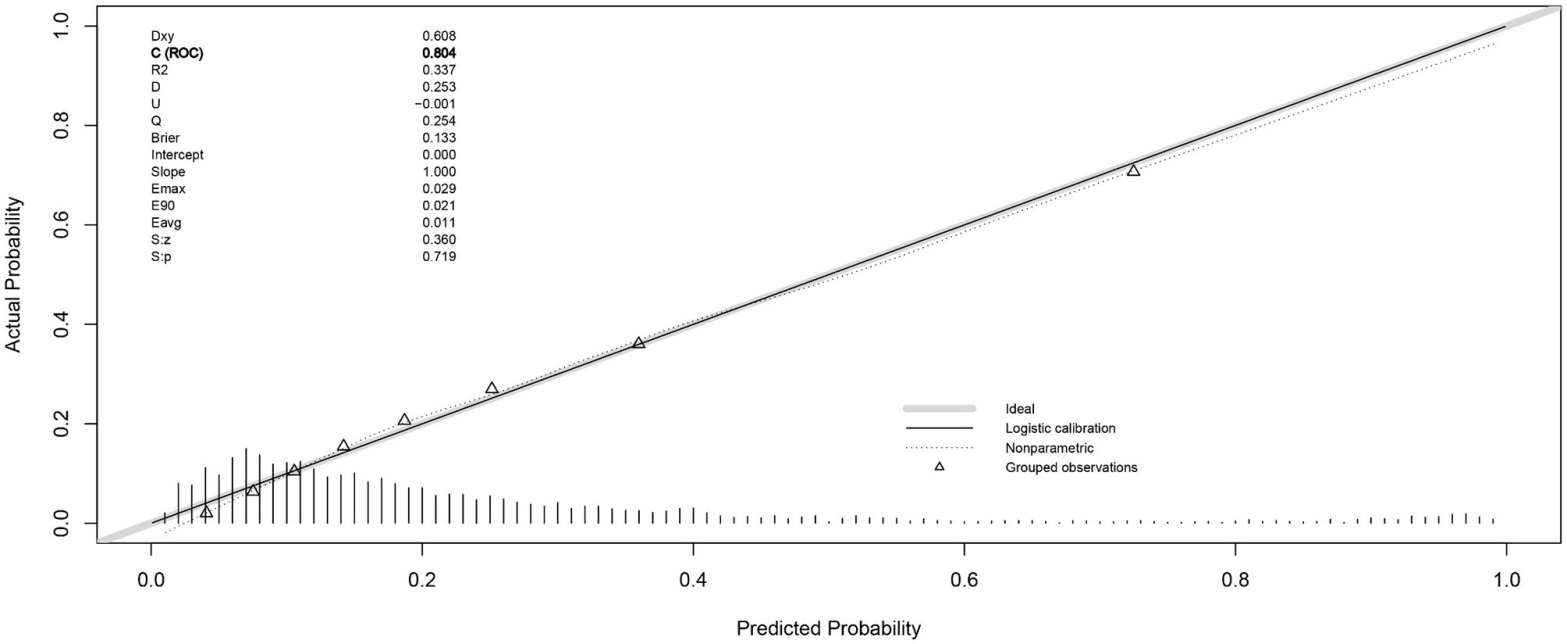
Cross-validated calibration plots of the prediction model in risk of smartphone addiction. The smaller distance of the scatter points from the dotted line, the better calibration indicated.

As shown in Figure 1, the result of logistic regression was visualized in the form of a nomogram. According to the calibration curve (Figure 2), the cross-validated C-index of the results of nomogram was 0.804, representing the predicted risk of smartphone addiction among children and adolescents was consistent with the observed incidence. Besides, we further identified that respondents who were female or unwilling to engage in medical profession were at greater risk of smartphone addiction.

**Figure 3:**
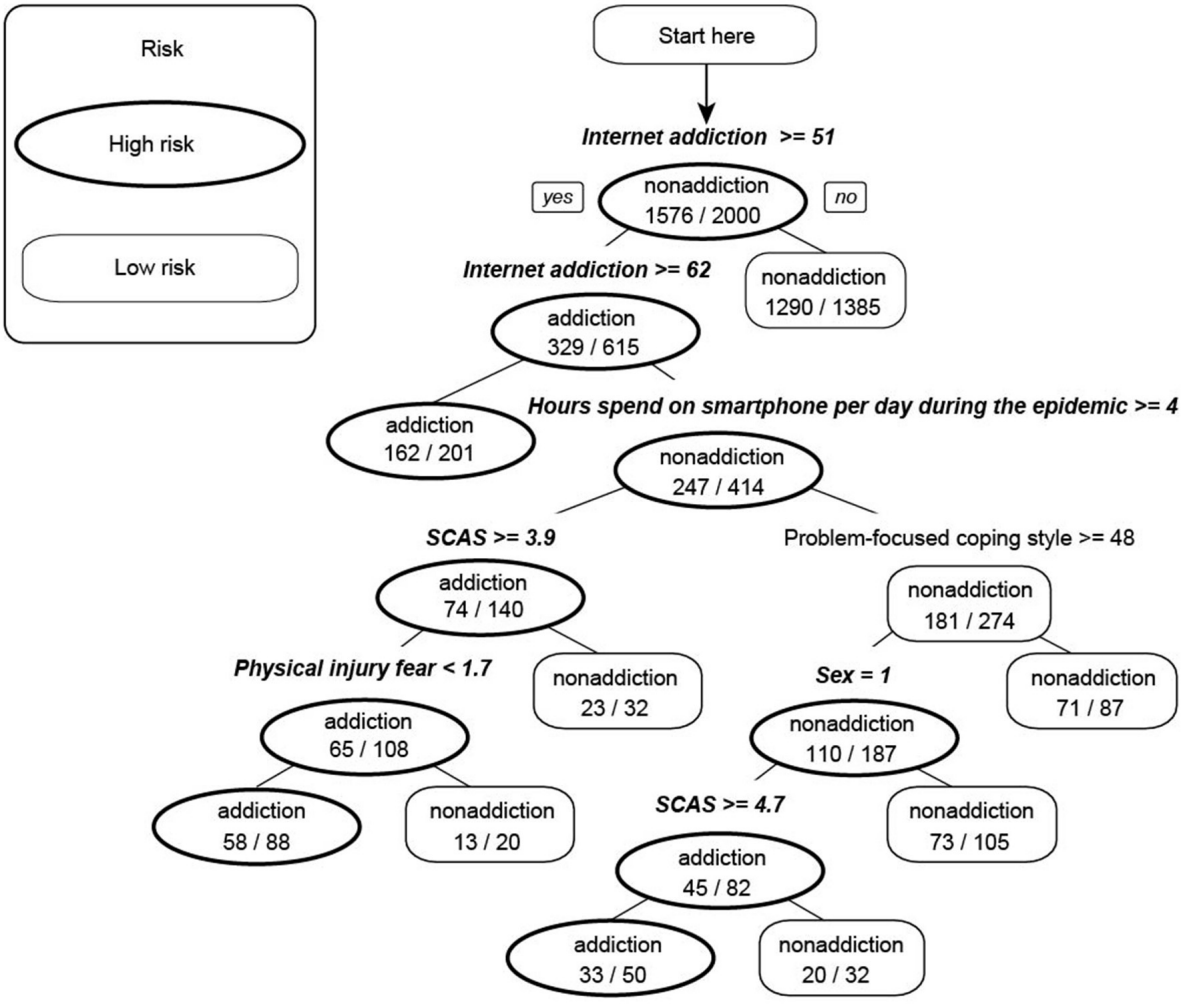
Decision tree for detecting smartphone addiction among children and adolescents. Anxiety symptoms mean the total scores of Spence Child Anxiety Scale. The assignment values of these classified variables were: sex, 1 = female; 2 = male; hours spend on smartphone per day during the epidemic, 1(≤1 h)−4 (≥5 h). Factors with significant meaning have been written in bold italic format.

The decision rules have illustrated in the decision tree model (Figure 3). Five variables were selected by the program for the decision tree of smartphone addiction. Among them, Internet addiction was the most significant determining factor, which located at the root of the decision tree and presented as the first-level split of two initial branches. Hours spend on smartphone during the epidemic (more than 5 h) and level of clinical anxiety symptoms (SCAS total scores) were located on the second-, third-level split, respectively. And sex (being female) and fear of physical injury were followed on the fourth-level. The classification accuracy, sensitivity, specificity, positive predictive value, negative predictive value, and area under the curve (AUC) for detecting smartphone addiction were 87.3, 71.4, 92.1, 73.5, 91.4%, and 0.884.

## Discussion

Due to its unstandardized diagnostic criterion (e.g., problematic phone use and smartphone addiction risk), the prevalence rates of children and adolescents vary widely from 5 to 50% (40). Therefore, in order to eliminate the bias caused by using different measurement and scoring criteria, we reviewed studies using the diagnostic scale of SAS-SV and found that the prevalence of smartphone addiction of children and adolescents was 22.8% in China (10), 16.9% in Switzerland (28), and 12.5% in Spain (41). These rates were all lower than the prevalence rates reported in our study (23.5%). We speculate that children and adolescents may be more at risk of smartphone addiction because they are yet to develop mature self-control and competence in smartphone use. This may particularly affect respondents whose family members were involved with pandemic work and may not receive sufficient care from their parents/caregivers, and therefore, able to use smartphones without guidance. Additionally, as a new generation of digital natives emerges with the curiosity to pursue novel experiences, respondents are desperate to express their thoughts, hunt for emotional support, and build peer relationships on a novel online stage using various applications (“Apps”) (42). The instant reactions and feedbacks delivered by smartphones further promote their dependence on smartphones (43). It was reported that netizens aged 15–19 years have the largest number of mobile apps per capita in China, and studying, listening to music and playing games were the most frequent online activities (1).

Furthermore, as shown in Table 1, the prevalence rate of smartphone addiction in children (24.8%) was slightly higher than adolescents (23.3%), which could be interpreted by the fact that Chinese students are encouraged to study hard and pursue better academic performance in a competitive learning atmosphere (44). As a result of the pandemic, the main method of studying was to take online classes. Adolescent in middle school were compelled to adapt to a faster pace of learning and bear greater academic pressure than children in primary schools, suggesting that they have to spend more time on studying instead of playing games online, which may somewhat reduce the risk of smartphone addiction.

Additionally, in order to develop a screening tool of smartphone addiction, we used three different statistical approaches, including logistic regression, a nomogram and decision tree. By plotting calibration, as well as calculating specificity, sensitivity, positive/negative predictive value, and AUC, results of logistic regression and model of decision tree were preferable tools to identify children and adolescents at high risk of smartphone addiction. According to the obtained logistic regression results and decision tree model (Figure 3), Internet addiction was the most significant variable, which was consistent with results of the previous studies (45). That was, based on the assumption of smartphone addition shares the same social and psychological properties (e.g., anxiety) with Internet addition, Choi et al. (45) proposed that excessive smartphone use (increased scores of smartphone addition) was significantly associated with higher level of Internet addiction, while the risk factors for Internet addiction also include uncontrolled smartphone use. The findings can also be explored by the fact that smartphones, with their instant access to the Internet at any place or time, large screen, and inherent mobility, can efficiently meet the needs of sociability, study, work, and other internet-based activities of subjects. Especially when they were quarantined at home, smartphone was the most convenient devices and Internet was the most important pathway to collection information or interact with the society. In recent years, uncontrolled overuse of smartphone, which can interfere with concentration at school or work (21.0% in Korea subjects), even cause physical difficulties (e.g., blurred vision, wrist or back pain, and sleep disturbances) has triggered numerous public concerns (46–48). Effective prevention and treatment should be utilized timely based on the predictive factors in clinical and education institutions.

Consistent with previous studies (49, 50), the results of nomogram analysis identified that girls were more likely to experience smartphone addiction (Figure 1). Evidence has shown that gender differences exist in mental health, in which girls show higher rates of mood disorders than do boys (51). Unlike their male counterparts who are more likely to be drawn to the functional purpose of smartphones, female students may be more likely to spend their time on social media, chatting, and texting, which may mitigate emotional distress to some extent (52). It further explains that females are more likely to be involved with their mobile devices (53) to alleviate various negative emotions during the epidemic, especially for smartphones which can be taken in hand and used anywhere at any time.

Regression results in Table 4 and Figure 2, as well as decision tree model in Figure 3 revealed that longer duration of daily usage of smartphones was associated with greater smartphone addiction. Previous work has reported that adolescents who spent more than 4 h a day demonstrated more problems in physical and psychological health (54). Cha et al. also recognized that the daily duration of smartphone is one of the most significant indicators of smartphone addiction (55). In China, 76% of male and 79.8% of female children (10–14 years of age) used smartphones, with this percentage as high as 98% among young adolescents (15–19 years). Moreover, the average time people spend on browsing the Internet on their mobile phones has reached 5.69 h per day, indicating a greater risk of smartphone addiction (1).

**Table 4:** Factors associated with the presence of smartphone addiction for respondents during the COVID-19 outbreak (*N* = 3,615).

**Variables**	***Unadjusted OR (95%CI)***	***Adjusted OR (95%CI)***
Sex	0.578 (0.475–0.703)	0.565 (0.465–0.686)
Hours spend on smartphone per day during the epidemic	1.541 (1.398–1.699)	1.544 (1.402–1.703)
Willingness to engage in medical profession	0.920 (0.856–0.989)	0.928 (0.863–0.997)
Anxiety symptoms	1.168 (1.098–1.243)	1.206 (1.152–1.264)
Physical injury fear	0.668 (0.536–0.832)	0.656 (0.527–0.816)
Internet addiction	20.167 (12.438–34.487)	21.438 (12.418–34.387)
Problem-focused coping style	0.987 (0.978–0.997)	0.987 (0.977–0.996)
Emotion-focused coping style	1.060 (1.049–1.073)	1.059 (1.047–1.071)

*Significant variables listed in Table 1 (the sociodemographic characteristics), Table 2 (contents of the COVID-related information), as well as levels of anxiety and its six dimensions, and subscales of coping style scale were used for performing logistic analysis by employing R software*.

*Anxiety symptoms mean the total scores of the Spence Child Anxiety Scale*.

*C-index of this adjusted model were 0.804*.

*COVID-19, coronavirus disease 2019; OR, odds ratio; CI, confidence interval*.

Anxiety has long been considered the most common mental health problem in children and adolescents (44). It may worsen by coping with negative consequences (e.g., disturbed learning and life style and financial strain) caused by the COVID-19 outbreak. In addition, patients infected with COVID-19 demonstrated functional impairment of respiratory, circulatory, digestive, and even loss of life in a short time (56), which may be a significant source of subjects' fear of anxiety and physical injury, further exacerbating their overall anxiety levels. Findings of this study were partially consistent with previous studies which assessed the relationship between psychological traits (e.g., anxiety and depression) and smartphone addiction (11, 57). In other words, anxiety symptoms have a positive relationship with problematic smartphone use severity (58). Evidence have suggested that the link between anxiety and smartphone addiction may not just exist within young adults (59). Children and adolescents may be unable to sufficiently manage their negative emotions (anxiety and depression), and may thus be highly susceptible to smartphone addiction (59). However, physical injury fear, as one subscale of the SCAS, found to be associated with the decrease of smartphone addiction. It can be speculated that smartphone allowed easy access for interpersonal communication and information-gathering, participants always carry it around when they go out or stay at home. Thus, inadequate or untimely disinfection of smartphone surfaces may increase the chance of being infected with the coronavirus. That is, the higher level of physical injury fear, the lower risk of smartphone addiction. Moreover, evidence suggests that parents experience potent, negative responses to COVID-19-related stressors (e.g., fear of infection, disruption to work, taking additional caregiving burden), representing symptoms of anxiety and posttraumatic stress (60). Referring to the theory of spillover hypothesis (61), the negative affect, mental or behavior disorder of parents can transfer with the same valence to children within the internal family system. This point of view will also one of the significant contents that we will verify in the subsequent studies.

Besides, problem-focused coping style was a significant factor leading to the occurrence of smartphone addiction among children and adolescents. The previous literature shows that coping strategies serve either a problem-focused coping function or an emotion-focused coping function (62). Individuals engaged in problem-focused coping strategies demonstrate that coping behaviors directly aimed at the source of the stress (63) and can prompt respondents to adopt positive coping styles to deal with adverse consequences caused by the pandemic. However, emotion-focused coping strategies denote the regulation of emotions that result from the stress. While children and adolescents are at a vulnerable developmental stage in emotion regulation, leading them to be more inclined to apply negative coping styles of endurance, avoidance, fantasy, and denial in dealing with stress (64), and it may therefore be an important risk factor for smartphone addiction.

Additionally, we found that participants who desired to engage in medical profession have lower risk of developing smartphone addiction. In this study, after the outbreak of epidemic, the proportion of participants who turned to consider medical profession as their ideal occupation was significantly higher than that of the uncertain population. Not to mention the number of participants who have been firmly determined to pursue medicine has vastly outnumbered those who have never considered a medically related career. With the approaching of the entrance or graduation examination, most Chinese students with specific career goals (e.g., medicine) were more focused on their studies instead of being indulge in smartphone. This is also consistent with previous studies in which higher academic achievement and increasing academic motivation seem to be negatively associated with addiction rates (49, 65, 66).

### Limitation

Several limitations must be considered when interpreting the findings of this study. First, the convenience sample approach limits the generalizability of the results. Second, the measures are based on children and adolescent self-report, particularly guardians participate in the answering process of elementary school students. These conditions might lead to potential confounding factors and implicit bias on underestimation or overestimation of participants' smartphone usage. In order to improve our findings, future studies should try to collect data from multiple informants (e.g., parents or other primary caregivers) using quantitative and qualitative (face-to-face interview) methods. Third, factors such as parenting style, personality trait, and sleep patterns may also influence respondents' smartphone addiction. Therefore, future studies should expand measurements and sample sources (especially samples from Hubei) to further improve the study design and explore the associations that we analyzed in this study.

## Conclusion

This study demonstrated that the prevalence rate of smartphone addiction among children and adolescents in mainland China is significantly higher during the pandemic. In addition, being female, duration of smartphone usage, levels of anxiety symptoms, and the type of coping strategies strongly influence risk for addiction. As the number of smartphone users grows in China, this study may shed light on the scale of smartphone addicts in the post-pandemic era and provide future scientific guidance for both policymakers in government departments and medical personnel in health institutions wishing to stem minors' dependency on smartphones. Moreover, as COVID-19 epidemic continuously deteriorates, the researchers believe that findings of this study will be beneficial to show the importance of the issue in the international arena and to aid educators and guardians in distinguishing between predictive factors for smartphone addiction and can consequently be utilized in the prevention and treatment.

## Data Availability Statement

The original contributions presented in the study are included in the article/supplementary material, further inquiries can be directed to the corresponding author/s.

## Ethics Statement

The studies involving human participants were reviewed and approved by the Ethics Committee of the First Affiliated Hospital of China Medical University (No. 2020-202-2). Written informed consent to participate in this study was provided by the participants' legal guardian/next of kin.

## Author Contributions

GZ designed and corrected this Paper. LD wrote the paper. LD, JH, ML, JD, and FL collected the data. All authors contributed to the article and approved the submitted version.

## Conflict of Interest

The authors declare that the research was conducted in the absence of any commercial or financial relationships that could be construed as a potential conflict of interest.
